# Treatment-resistant obsessive-compulsive disorder: pharmacological augmentation and neuromodulation - effectiveness and safety

**DOI:** 10.1192/j.eurpsy.2026.10640

**Published:** 2026-07-31

**Authors:** B. Mihaela, D. Jelaga

**Affiliations:** Department of Mental Health, Medical Psychology and Psychotherapy, Nicolae Testemițanu State University of Medicine and Pharmacy, Chisinau, Moldova, Republic of

## Abstract

**Introduction:**

Approximately 40–60% of adults with OCD remain symptomatic after optimal SSRIs (and clomipramine) plus exposure and response prevention. Evidence for augmentation and neuromodulation expanded, yet effect sizes and safety remain heterogeneous.

**Objectives:**

(1) Quantify effectiveness and safety of pharmacological augmentation in adults with treatment-resistant OCD; (2) synthesise outcomes for deep transcranial magnetic stimulation, transcranial direct current stimulation and deep brain stimulation; (3) identify optimisation signals (dose, duration, targets).

**Methods:**

Systematic searches in MEDLINE/PubMed, Embase, PsycINFO and Web of Science (2020–2025) in adults with TR-OCD. Designs: RCTs, meta-analyses, observational safety cohorts. Comparators: placebo/treatment-as-usual (drugs), sham (devices). Outcomes: Y-BOCS change, response (≥35%), AE/SAE, discontinuations; metrics: RR, OR, NNT. Flow: 1,004 records; 742 post-deduplication; 114 full texts; 26 included; 88 excluded. Populations: mean age 30–45; women 45–55%; tertiary clinics. Mixed samples included only with TR-OCD subgroup data or ≥70% TR; other effects interpreted cautiously.

**Results:**

Antipsychotic augmentation: response 33% in SSRI nonresponders (NNT≈5); risperidone/aripiprazole consistent; quetiapine/olanzapine inconsistent. AE: akathisia 10–20%, sedation/weight gain 15–30%; discontinuations 10–25%. Glutamatergic: memantine (7 RCTs; n=315) large effect by ≥8 weeks (SMD≈−1.17; 20 mg/day); rash risk ↑ (RR≈7.00); discontinuations 0–3%. N-acetylcysteine (6 RCTs; n=195) modest improvement at 5–8 weeks (≈−3 Y-BOCS points); AE ~placebo (0–2%); durability >12 weeks not consistently reported. 5-HT3 antagonists (6 RCTs; n=334; ondansetron/granisetron/tropisetron) reduced Y-BOCS total/subscales; discontinuations 0–2%; AE mild, mainly gastrointestinal. Neuromodulation: dTMS (4 RCTs; n=252) RR 3.71 post-treatment and 2.60 at 1 month; absolute gain +20–30 pp; AE: headache 35–38%, local discomfort ~30%; seizures <0.01%/session. Real-world: 72.6% initial; 52.4% ≥1 month. tDCS (7 RCTs; n=201) small–moderate effect (SMD≈−0.46); AE ~sham; durability >1–3 months not consistently reported. DBS (9 RCTs; n=91) −5.1 Y-BOCS vs sham; OR≈4.7; NNT≈3.9; assessed ≥3–6 months; hardware ≈8%, infections 4–5%, hypomania/suicidality 3–4%.

**Conclusions:**

Antipsychotic augmentation yields ~33% response with tolerability costs. Glutamatergic add-ons: memantine (large effect; rash risk), N-acetylcysteine (modest, time-dependent; safe), 5-HT3 antagonists (consistent; discontinuations 0–2%). Among devices, dTMS shows most consistent short-term evidence (RR 3.71; +20–30 pp; 52% sustained); tDCS gives small–moderate gains; durability uncertain; DBS effective for severe, selected cases (NNT≈3.9). Major limitation: between-study heterogeneity in TR-OCD definitions and stimulation/augmentation parameters limits interpretability and generalisability.

**Disclosure of Interest:**

None Declared

